# Role of complement activation in anti-neutrophil cytoplasmic antibody-associated glomerulonephritis

**DOI:** 10.3389/fmed.2022.1031445

**Published:** 2022-12-02

**Authors:** Tadasu Kojima, Takashi Oda

**Affiliations:** Department of Nephrology and Blood Purification, Kidney Disease Center, Tokyo Medical University Hachioji Medical Center, Hachioji, Japan

**Keywords:** anti-neutrophil cytoplasmic antibody (ANCA), complement activation, rapidly progressive glomerulonephritis, alternative pathway, classical pathway, lectin pathway, avacopan, eculizumab

## Abstract

Anti-neutrophil cytoplasmic antibody (ANCA)-associated vasculitis (AAV) is an autoimmune disease characterized by necrotizing inflammation of small or medium vessels, causing ANCA associated glomerulonephritis (AAGN). AAGN is defined as pauci-immune glomerulonephritis with no or little immune deposition; hence, activation of the complement system in AAV was overlooked until recently. However, many studies in mice and humans have revealed a crucial role for complement system activation in the development of AAGN. Circulating and urinary detection of various complement components associated with AP activation, which have been broadly correlated with the clinical activity of AAGN, has been reported and may be useful for predicting renal outcome at the time of diagnosis and setting up personalized treatments. Moreover, recent investigations have suggested the possible contribution of the complement classical or lectin pathway activation in the development of AAGN. Thus, as therapeutic options targeting complement components are making rapid strides, the primary complement pathway involved in AAGN disease progression remains to be elucidated: this will directly impact the development of novel therapeutic strategies with high specificity and reduced side effects. This review summarizes and discusses the most recent evidence on the crucial roles of the complement system in the development of AAGN and possible therapeutic strategies that target complement components for disease management.

## Introduction

Anti-neutrophil cytoplasmic antibody (ANCA)-associated vasculitis (AAV) is a systemic autoimmune disease associated with small-vessel inflammation in various organs, especially the kidney (glomerulonephritis) and lungs (pulmonary capillaritis), with or without granulomatosis in the upper and lower respiratory tracts. AAV is categorized into three diseases based on the absence or presence of granuloma formation and marked eosinophilia, namely, microscopic polyangiitis, granulomatosis with polyangiitis, and eosinophilic granulomatosis with polyangiitis (1). AAV that affects the kidney as glomerulonephritis is called ANCA-associated glomerulonephritis (AAGN). AAGN is clinically significant owing to its manifestation as rapidly progressive glomerulonephritis with poor renal prognosis as well as increasing worldwide prevalence among the aging population (2).

The pathogenesis of AAGN may be divided into two steps: initiation step (an initial insult or event that cause disease development) and promotion step (the factors that induces initial change into established disease state). The mechanism of AAGN initiation is poorly understood, but genetic, immune, and environmental factors, including infections, microparticles, and drugs (3–5) may play crucial roles. However, recent research has shed some light on the mechanism underlying AAGN promotion.

Anti-neutrophil cytoplasmic antibody is an autoantibody against cell components of neutrophils. It primarily targets two antigens, myeloperoxidase (MPO) and proteinase 3, which exist in the cytoplasm of neutrophils under normal physiological conditions; however, exposure to proinflammatory cytokines, such as interleukin-1 beta and tumor necrosis factor alpha, stimulates translocation of the antigens to the cell surface (6). After binding of ANCAs to antigens on the cell surface, neutrophils are activated, leading to the release of enzymes, reactive oxygen species, proteases, and neutrophil extracellular traps (NETs), which potentially cause vasculitis (7–9). Thus, ANCA is not only a biomarker, but also one of the most essential factors leading to the promotion of AAV.

Treatment for patients with AAGN consists of induction of remission therapy to suppress inflammation and reduce renal scarring, and maintenance therapy to prevent disease relapses. The KDIGO 2021 Clinical Practice Guideline for the Management of Glomerular Diseases recommend the use of glucocorticoids in combination with cyclophosphamide or rituximab as remission therapy for new-onset AAGN, and the use of low-dose glucocorticoids with rituximab or azathioprine as maintenance therapy after remission (10). The developments in immunosuppressive therapy have led to a better prognosis (11), however, infections resulting from treatment adverse effects of broad immunosuppression remain a major cause of deaths (12, 13), and therefore treatment strategies having similar therapeutic effects but with more specific and limited immunosuppression is strongly desired.

The 2012 Chapel Hill Consensus Conference Nomenclature of Vasculitides (14) defines AAGN as a pauci-immune necrotizing crescentic glomerulonephritis, which is characterized by little or no deposition of immunoglobulins or complement components in glomeruli. Moreover, owing to the rarity of hypocomplementemia in AAGN patients, the immunoglobulin and complement activation reactions have not been suspected to play a major role in the development of AAGN. However, in recent decades, increasing evidence suggests the role of complement activation in AAGN pathogenesis (15, 16). Based on these findings, C5a is one of the therapeutic targets which is clinically used both for remission induction and maintenance of AAGN. In this review, we briefly summarize the recent evidence for the involvement of the complement activation and its possible implications in AAGN management.

## Overview of the complement system

**FIGURE 1:**
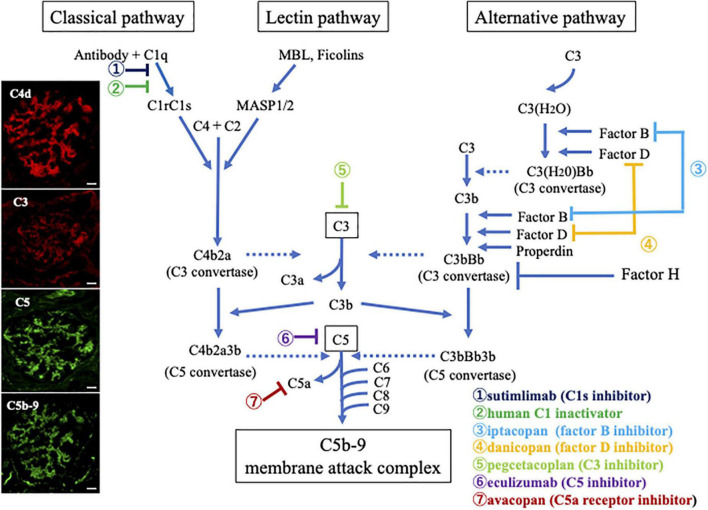
Overview illustration of the complement activation system with therapeutic targets, and representative photomicrographs of the deposition of complement breakdown products in glomeruli of a patient with myeloperoxidase (MPO)-anti-neutrophil cytoplasmic antibody (ANCA)-associated glomerulonephritis (AAGN). Photographs on the left side show immunofluorescence staining for C4d (Alexa Fluor 594: red), C3 (Alexa Fluor 594: red), C5 (Alexa Fluor 488: green), and C5b-9 (Alexa Fluor 488: green) in a renal biopsy specimen of a patient with MPO-AAGN (Original magnification:×400, Scale bars = 20.0 μm in all photographs). The positive staining results suggest that immune complexes activate the classical complement pathway, leading to the activation of the final common pathway and formation of C5b-9 in patients with MPO-AAGN. Outlines of other pathways, including the lectin and alternative pathways and possible therapeutic targets are also summarized.

The complement system is comprised of over 50 proteins, most of which are primarily produced in the liver (17), although a high concentration of these proteins is found in certain locations, such as the kidneys. Being an integral part of the innate immune system, this system plays a key role in the clearance of foreign substances, especially microbes. It is activated *via* three pathways: the classical, lectin, or alternative pathway, as outlined in Figure 1. The classical pathway is triggered by the IgM or IgG antigen/antibody complexes that bind to C1 (18). The C1 complex consists of three subcomponents: C1q, C1r, and C1s. When C1q binds to immune complexes, it causes a structural change that activates C1r, which then divides and activates C1s (19, 20). Subsequently, activated C1s cleaves downstream components, C4 and C2, and forms the classical C3 convertase (C4b2a). Of note, the lectin pathway is initiated by the binding of plasma molecules, such as mannose-binding lectin (MBL), ficolins, and collectin, to bacterial surfaces with mannose-containing polysaccharides, which results in the association of two serine proteases, MBL-associated serine proteases (MASP)-1 and MASP-2. The MBL/MASP-1/MASP-2 complex activates MASPs which cleave C4 into C4a and C4b and C2 into C2a and C2b, respectively, thus forming C4b2a (21). Therefore, these pathways share C2 and C4 components during the formation of C3 convertase. In contrast, the alternative pathway is activated upon spontaneous hydrolysis of C3, which leads to the formation of C3(H_2_O). C3(H_2_O) binds to the serine protease factor B, which in turn is cleaved by the serine protease factor D to form Ba and Bb, forming C3(H_2_O)Bb which cleaves C3 into C3a and C3b (22). C3b binds with factors B and is cleaved by factor D into Ba and Bb, forming alternative C3 convertase (C3bBb). C3 convertases from every complement activation pathway activate C3, breaking it down into C3a and C3b. The latter binds to C2a and C4b to form C5 convertase. The C5 convertase is C4b2a3b in the classical or lectin pathways, and C3bBb3b in the alternative pathway. C5 is broken down into C5a and C5b by C5 convertase. C5b then forms the membrane attack complex (MAC) with complement components C6-C9. Of note, C3a, C5a, and MAC are important inflammatory mediators of the complement system. C3a and C5a (anaphylatoxin) bind to receptors on the surface of target cells, such as macrophages, neutrophils, and endothelial cells, and release proinflammatory cytokines and vasoactive agents. The MAC insert into the membrane of the target cell and form functional pores that lead to cell lysis and death (23, 24). Importantly, the complement system is regulated by inhibiting proteins, such as C1 inhibitor, factor H, factor I, membrane cofactor protein, and decay acceleration factor.

As mentioned above, the complement system plays a crucial role in the normal physiological function of defense against infection, but its pathological activation contributes to the development of autoimmune disorders, such as atypical hemolytic uremic syndrome, C3 glomerulopathies, immune complex membranoproliferative glomerulonephritis, systemic lupus erythematosus, and AAV (25–28).

## Complement system and ANCA-associated glomerulonephritis

### Complement deposition in renal tissue

Owing to its diagnostic classification as a pauci-immune glomerulonephritis, involvement of the immune complex in AAGN has rarely been reported. However, some studies report the presence of glomerular immune-complex deposits in renal biopsies of patients with AAGN (29–31): complement deposits in kidney biopsies have been reported in 25–54% of patients with AAGN.

We analyzed the deposition of complement components in the renal tissues of 20 patients, who were positive for MPO-ANCA and diagnosed with nephritis in association with MPA but without any other nephritis, such as anti-glomerular basement membrane diseases or IgA nephropathy, and reported the marked deposition of various components in glomeruli (32). As shown in Table 1, staining for pan-specific complement C3 and C5 was positive in 35 and 60% of patients, respectively, if the staining intensities were classified into five levels (− to +++), and intensities of + or higher were defined as positive. On the same scale, C4d and C5b-9 were positive in 75 and 85% of the patients, respectively. On the other hand, only 20, 5, and 0% of the patients were positive for factor Bb, both MASP-1 and MASP-2, and MBL, respectively. Thus, we found definite glomerular deposition of C4d, C5, and C5b-9 in AAGN patients (Figure 1; extreme left corner). In another report, Oba et al. reported that C3 deposition was found in 39.4% of AAGN patients, and deposition of C3 had a lower serum C3 levels and poor renal and overall survival rates (33). Lin et al. (34). analyzed immune deposits in 97 patients with AAGN and found that C3 staining was seen in 18.56% cases, C1q in 10.75%, IgA in 18.75%, IgM in 17.71%, and IgG in 13.54%. Furthermore, patients with IC deposition had increased proteinuria and serum creatinine levels and lower platelet counts, and serum C3 and IgG levels than patients with pauci-immune deposition at the time of diagnosis. Additionally, immunoglobulin levels in the kidney specimens were accompanied by C3 deposition, and IgG deposition was associated with a high rate of end-stage kidney disease, while IgA deposits were associated with granuloma-like and vascular lesions in AAGN. Another study showed that electron-dense deposits were also found in more than 50% of the renal specimens from AAGN patients (29).

**TABLE 1:** Results of immunofluorescence staining in 20 patients with ANCA associated glomerulonephritis (AAGN).

CASE	IgG	IgA	IgM	C1q	C3c	panC3	C5	C4d	C5b-9	factor Bb	MASP-1	MASP-2	MBL
1	**−**	**−**	**−**	**−**	**−**	**−**	**±**	**+**	**+**	**+**	**−**	**−**	**−**
2	**−**	**−**	**−**	**−**	**−**	**+**	**+**	**+**	**+**	**+**	**+**	**−**	**±**
3	**−**	**−**	**−**	**−**	**−**	**+**	**++**	**++**	**++**	**±**	**−**	**−**	**−**
4	**−**	**−**	**−**	**−**	**−**	**±**	**±**	**+**	**+**	**±**	**−**	**−**	**−**
5	**−**	**−**	**−**	**−**	**±**	**+**	**+**	**±**	**+**	**±**	**−**	**−**	**−**
6	**±**	**±**	**±**	**±**	**±**	**±**	**+++**	**++**	**++**	**−**	**−**	**−**	**−**
7	**−**	**−**	**+**	**±**	**+**	**±**	**+**	**+**	**+**	**−**	**−**	**−**	**±**
8	**−**	**−**	**−**	**−**	**±**	**±**	**+**	**+**	**+**	**±**	**−**	**−**	**−**
9	**−**	**−**	**−**	**+**	**−**	**+**	**±**	**−**	**+**	**+**	**±**	**−**	**−**
10	**+**	**+**	**+**	**−**	**−**	**±**	**±**	**++**	**+**	**−**	**−**	**−**	**±**
11	**±**	**−**	**−**	**±**	**±**	**±**	**++**	**+**	**+**	**−**	**±**	**±**	**−**
12	**−**	**−**	**+**	**−**	**−**	**±**	**±**	**+**	**±**	**−**	**−**	**−**	**−**
13	**−**	**−**	**±**	**−**	**−**	**±**	**±**	**±**	**±**	**±**	**±**	**+**	**−**
14	**−**	**±**	**−**	**±**	**±**	**±**	**±**	**±**	**±**	**+**	**−**	**−**	**±**
15	**−**	**−**	**−**	**−**	**−**	**−**	**+**	**+**	**+**	**±**	**−**	**−**	**−**
16	**−**	**−**	**+**	**±**	**±**	**+**	**+**	**+**	**+**	**−**	**−**	**−**	**±**
17	**−**	**−**	**−**	**−**	**−**	**−**	**±**	**±**	**+**	**−**	**−**	**−**	**−**
18	**−**	**−**	**−**	**−**	**+**	**++**	**++**	**+++**	**++**	**±**	**±**	**−**	**−**
19	**−**	**−**	**+**	**+**	**−**	**±**	**++**	**+++**	**+**	**−**	**−**	**−**	**−**
20	**−**	**−**	**−**	**−**	**±**	**+**	**+**	**+**	**++**	**−**	**−**	**−**	**−**
positive rate (%)	5	5	25	10	10	35	60	75	85	20	5	5	0

All of the glomerular immunofluorescence staining results were semi-quantitatively classified into five grades, as −, ±, +, ++, and +++, and intensities higher than + were defined as positive.

These findings suggest that immune complex deposition is associated with clinicopathological features of AAGN. Notably, immune complex deposition in AAGN is not uncommon, as previously considered. Hilhorst et al. speculated that immune complexes are not detected by electron microscopy in patients with AAV because they might be rapidly degraded in the early phase of clinical course (35).

On the other hand, Gou et al., reported that the deposition of the alternative complement activation product of Bb in glomeruli of patients with AAGN, which correlated with percentage of total crescents, degree of interstitial infiltration, fibrosis, and tubular atrophy (36).

### Role of alternative pathway activation

Recent evidence from a murine model of MPO-AAV points to a critical role of the complement system, especially the alternative complement pathway. In 2002, Xiao et al. established a murine model showing the direct pathogenic role of ANCA in MPO-AAV; injection of anti-MPO IgG from MPO-deficient mice immunized with murine MPO into immunodeficient mice or wild-type mice definitely induced pauci-immune necrotizing and crescentic glomerulonephritis (NCGN) (37). In this antibody-transfer NCGN mouse model, complement depletion by cobra venom factor completely blocked the development of NCGN. In addition, C5 knockout mice did not develop NCGN, but C4 knockout mice did (16). Low levels of circulating C3 have been reported in 4–35% of AAV patients (31, 38–44). Interestingly, low levels of serum C3, but not C4, can be a predictor of renal outcome at diagnosis. Gou et al. reported that the plasma levels of Bb in AAV patients were correlated with disease activity, such as erythrocyte sedimentation rate, Birmingham vasculitis activity score and crescent formation rate on renal biopsy (36). Hillhorst et al. reported complement deposition of C3d and/or properdin in the patients with AAGN were associated with cellular crescents and proteinuria, suggesting complement activation in AAGN occur predominantly *via* alternative pathway (35).

Sethi et al. evaluated the protein in glomerular depositions using laser microdissection and mass spectrometry. They found low spectra numbers for C3 and immunoglobulins, and minimal or no C4 and C9 in patients with AAGN. Thus, the spectra numbers for C3 were higher than those for C4 in AAGN, indicating activation of the alternative complement pathway (45). The alternative pathway can be activated by NETs, including properdin and factor B (46). Properdin, a plasma glycoprotein which acts as a positive regulator of the alternative pathway, contributes to the progression of renal damage (47, 48). By contrast, Chen et al. showed that plasma levels of complement factor H, a negative regulator of the alternative complement pathway, were inversely associated with the activity of AAV, including the Birmingham vasculitis activity score (BVAS), serum creatinine level, and crescent formation rate in glomeruli (49). Thus, factor H deficiency may amplify the positive feedback loop of neutrophil activation and the alternative pathway, contributing to AAV severity.

### Role of classical and lectin pathway activation

Lin et al. evaluated histopathologic characteristics of patients with AAV and showed that deposition of C3 and C1q were not uncommon, suggesting that not only the alternative pathway, but also the classical pathway is involved in AAGN (34). We recently reported that 65% of patients with AAV had positive results for circulating immune complexes detected by the monoclonal rheumatoid factor assay using an ELISA kit (32). In addition, there was a positive correlation between the levels of circulating immune complexes and the serum C5a and C5b-9 levels measured by ELISA. Furthermore, C4d, C5, and C5b-9 deposition was seen in similar distributions by immunofluorescence in patients with AAGN (Figure 1). These results suggest that immune complexes trigger the classical pathway activation, which leads to activation of the common pathway that continues through C5. In contrast, Yoon et al. reported that serum MBL is correlated with BVAS and pulmonary manifestation score in patients with AAV (50). Additionally, the levels of urinary MBL and C1q in patients with AAV are significantly higher than those in healthy controls, supporting the concept that classical/lectin pathways contribute to the progression of AAV.

Gou et al. reported that the levels of circulating C4d in AAGN patients were significantly higher than those in controls, however, the levels of circulating C4d did not change between active phase and remission phase in AAGN patients (36). From this point of view, circulating C4d seems to be involved in the pathogenesis of AAGN, but it would not indicate disease activity.

Thus, there are limited amount of evidence regarding the classical and lectin pathway in AAGN compared to the alternative pathway activation.

### Roles of C5a, C5a receptor

The importance of C5a and its receptor on neutrophil in patients with AAV has recently become apparent (51). Activation of the complement pathway produces C5a, which induces ANCA antigen translocation through recruitment of p38 mitogen-activated protein kinase, phosphoinositol 3-kinase, protein kinase C, and extracellular signal-regulated kinase (52, 53). In the MPO-ANCA-induced NCGN model in mice, bone marrow transplantation from C5a receptor (C5aR)-deficient mice significantly protected them from developing NCGN (54). Thus, C5aR was required for the development of glomerulonephritis in this model. Furthermore, Xiao et al. evaluated the effect of genetic deficiency of C6 in a mouse model of anti-MPO antibody-induced NCGN and found no effect on the severity of the disease (55). Therefore, they claimed that C5a was essential, but C6 or C5b-9 were not crucial for the development of AAGN. They also found that another C5aR, C5aR-like 2 (C5L2), had properties opposite to C5aR. C5L2 knockout mice showed increased disease severity when injected with anti-MPO IgG, indicating that C5L2 engagement suppresses inflammation.

Consistent with these findings, Yuan et al. reported elevated plasma and urinary C5a levels in human AAGN, indicating remarkable complement activation in this disease (15). Furthermore, in AAV patients, a recent meta-analysis showed that the levels of serum C3a, C5a, C5b-9, and Bb were higher in the active phase than those in the remission phase (56). In addition, Hakroush et al. showed that C3c contributes to tubulointerstitial injury through interstitial vasculitis in AAGN (57).

### Complement-targeted therapies in ANCA associated glomerulonephritis

The current treatment options for AAGN are centered around high-dose glucocorticoids, often in combination with other immunosuppressants, such as rituximab or cyclophosphamide. Such treatment strategies significantly improved the prognosis of AAGN (11), however, these treatments have various adverse effects especially those related with infections resulting from broad immunosuppression (12, 13); treatment strategies having similar therapeutic effects but with more specific and limited immunosuppression is strongly desired.

As described above, complement system involvement in AAGN was first reported in mice with MPO-ANCA-induced NCGN, showing that activation of the alternative pathway, especially C5a, is essential for disease development. Neutrophils activated by ANCA release NETs and properdin that activate the alternative complement pathway and promote C5a production. C5aR is expressed on neutrophils, and binding of C5a to C5aR results in neutrophil priming. These findings indicate that the C5 or C5a-C5aR interaction may be a therapeutic target for AAGN. Eculizumab is a monoclonal antibody which binds to C5 and inhibits the cleavage of C5 into C5a and C5b, thereby, blocking subsequent complement activation, leading to MAC formation. Initially, this drug was used to treat atypical hemolytic uremic syndrome in the field of nephrology. It produced impressive results, with a reduction in end-stage kidney disease from 50% in 1-year historical cohorts to 6–15% post-treatment (58–61). Therefore, it is suspected to be a possible candidate for AAV treatment. According to our immunofluorescence staining results of 20 AAGN patients, definite C5 deposition was observed in the glomeruli of 60% of AAGN patients (Table 1 and Figure 1). For the histological staining of C5, we used the drug of eculizumab (anti-C5 monoclonal antibody, Alexion Pharmaceuticals) after conjugation of fluorescent dye, the positive results suggested the existence of C5 molecules with binding sites for this drug (the portion inducing dissociative activation of C5) in glomeruli; this may be direct evidence of the efficacy of eculizumab against AAGN (32). Indeed, case reports showed that eculizumab was effective in remission induction of active AAV (62). Moreover, Kitamura et al. reported a case of secondary thrombotic micro angiopathy (TMA) with posterior reversible encephalopathy syndrome and RPGN due to severe MPA who achieved remission after two doses of eculizumab, suggesting the usefulness of eculizumab for the treatment of TMA secondary to severe AAGN resistant to plasmapheresis (63). However, blocking the MAC formation involving C5b, partially inactivates innate immunity, especially against polysaccharide-encapsulated bacteria, such as *Neisseria meningitidis*, which increases the risk of invasive meningococcal infection. Thus, C5 blockade requires careful monitoring and anti-meningococcal vaccination to reduce the risk of infection. Furthermore, according to the findings in an experimental mouse model, C6 knockout had little impact on the disease severity of anti-MPO antibody-induced NCGN, thus showing that C5a was essential, but C6 or C5b-9 was not crucial for the development of AAGN. In addition, the inhibition of whole C5 molecules may also suppress the expected anti-inflammatory effect of C5L2 (55). From a drug safety aspect, treatment targeting C5a seems to be more appropriate than that targeting whole C5 in patients with AAGN.

According to recent reports from a phase III study (ADVOCATE) (64), an oral C5aR-antagonist, avacopan, is a safe and effective replacement for glucocorticoids in the induction and maintenance therapy for AAV. In this study, 331 patients with new or relapsing AAV were randomized to either high-dose prednisone tapered and discontinued by week 21 or avacopan for 52 weeks, after screening period for trial eligibility. Screening period was not to exceed 2 weeks, and glucocorticoid therapy had to be tapered to 20 mg or less of prednisone equivalent before the trial. All participants received cyclophosphamide followed by azathioprine or rituximab. BVAS remission at week 26 was achieved in 72.3% (120/166) of patients in the avacopan group and 70.1% (115/164) of patients in the prednisone groups, respectively (non-inferiority). Sustained remission at week 52 was achieved in 65.7% (109/166) of patients in the avacopan group and 54.9% (90/164) of patients in the prednisone groups, respectively (superiority of avacopan). Relapses rate at week 52 was lower in the avacopan group (10.1%) than in the prednisone group (21%), although rapid weaning off of glucocorticoids may have been related to this result. Moreover, in the subgroup analysis of this study, the least-squares mean change at week 52 from baseline of eGFR was higher in the avacopan group at 52 weeks than in the prednisone group (7.3 vs. 4.1 ml/min/1.73 m^2^). For the least-squares mean for the Glucocorticoid Toxicity Index-Cumulative Worsening Score at week 26, the avacopan group was significantly lower compared to the prednisone group (39.7 vs. 56.6). Moreover, for the least squares mean for the Glucocorticoid Toxicity Index-Aggregate Improvement Score at week 26, the avacopan group was also significantly lower than in the avacopan group than in the prednisone group (11.2 vs. 23.4). The incidence of adverse events possibly due to glucocorticoids on the basis of European League against Rheumatism criteria was significant lower in the avacopan group (66.3%) than in the prednisone group (80.5%).

In summary, avacopan is an important breakthrough as the first established anti-complement therapy available for use in the clinical field of AAV. It is an alternative to glucocorticoids and has less adverse effects. In 2021, avacopan was approved for the treatment of patients with AAV by the U.S. Food and Drug Administration.

Considering the recent development of various drugs targeting certain molecular components that block specific steps of the complement pathways other than avacopan or eculizumab, such as sutimlimab, C1 inactivator and pegcetacoplan, iptacopan, and danicopan, which inhibit C1s (65), C3 (66, 67), factor B (68), and factor D (69), respectively (Figure 1), the elucidation of the precise complement pathways related to the pathogenesis of AAGN should be clarified, as it would directly impact therapeutic efficiency.

## Conclusion

The role of the complement system in the pathogenesis of AAGN has been neglected for a long time, but evidence of its contribution, especially those related to the alternative complement pathway, has recently accumulated. Current treatment strategies for AAV are based on broad immunosuppression, which may be associated with a high risk of adverse effects. However, advances in the understanding of the precise pathogenic process related with complement activation of this disease are revealing new treatment strategies with fewer adverse effects. Further studies should be conducted to elucidate the clinical role of complement activation in AAGN and the use of novel therapeutics with more favorable outcomes.

## Author contributions

TK wrote the first draft. TO critically reviewed and revised the manuscript. Both authors have conducted literature searches related to the research topic, read, and agreed to the published version of the manuscript.

## Abbreviations

ANCA: anti-neutrophil cytoplasmic antibody
AAV: ANCA-associated vasculitis
AAGN: ANCA-associated glomerulonephritis
MPO: myeloperoxidase
NETs: neutrophil extracellular traps
MBL: mannose-binding lectin
MASP: MBL-associated serine proteases
MAC: membrane attack complex
NCGN: necrotizing and crescentic glomerulonephritis.
